# Menu labeling implementation in dine-in restaurants: the Public’s knowledge, attitude and practices

**DOI:** 10.1186/s13690-017-0177-9

**Published:** 2017-02-27

**Authors:** Hadia Radwan, Eman M. Faroukh, Reyad Shaker Obaid

**Affiliations:** 1 Department of Clinical Nutrition and Dietetics, College of Health Sciences/Sharjah Institute for Medical Research, University of Sharjah, P. O Box 27272, Sharjah, United Arab Emirates; 2 Dubai Health Authority, Dubai, United Arab Emirates

**Keywords:** Menu labeling, Nutrition, Restaurants, Knowledge, Attitude, Practice

## Abstract

**Background:**

The practice of menu labeling is gaining popularity worldwide as a potential policy to reduce energy intake as a means to decrease the prevalence of obesity. So the purpose of the present study is to identify the knowledge, attitudes, and practices of adults regarding the implementation of menu labeling in dine-in restaurants.

**Methods:**

A cross sectional survey included 2020 male or female adults (aged ≥ 18 years old) participants was collected from two cities in the United Arab Emirates(UAE). The participants filled a validated questionnaire in public places in two cities. A chi-squared test was conducted to compare responses for differences in proportions.

**Results:**

Most participants were knowledgeable about energy requirements for moderately active men (60%) and women (59%), but underestimated energy requirements for inactive adults (34%). The majority of the respondents favored the requirement to post calorie information on menus of dine-in restaurants at the point of purchase (76%). About half the respondents (48%) were more likely to visit restaurants with labeled menus.

**Conclusion:**

The results from this study may form the basis for future strategies in mandating calorie labeling of restaurant menu items in UAE. Menu labeling may be a useful policy tool for promoting appropriate caloric consumption.

## Background

Excessive eating and unhealthy food selections are causative factors in one of the most persistent health apprehensions facing the Gulf countries. The latest research investigating the prevalence of obesity reveals that approximately one quarter of adults in the Gulf countries, 28.52% of women and 15.5% of men over the age of 20, are obese [1]. According to a recent report from the Health Authority in Abu Dhabi, obesity may account for up to 60% of nationals in the United Arab Emirates (UAE) [2]. An area of emerging importance as a policy with the potential to reduce the widespread occurrence of obesity is the display of calorie information on restaurant menus in both dine-in and fast food chain restaurants [3].

Menu labeling is one strategy among a broad spectrum of efforts to reduce rates of obesity and its co-morbidities. Studies have shown that 90% of individuals ordering from restaurants underestimate the amount of energy within meals by as much as 600 kcal [4]. In the United States, a federal law obligates restaurant chains with 20 or more outlets to post nutrition information on their menus [5, 6]. Many studies have shown that the public is interested in knowing the number of calories present in the meals they order from restaurants [7]. Moreover, Spicer [8] showed that promoting the use of calorie information, and calorie awareness, supports lower-calorie choices at fast food eateries. Caloric labeling is proposed as an innovative approach that will change the food environment, and increases customers’ awareness of calories, which may, in turn, help to lower the costs of the obesity epidemic [9].

Energy-dense foods consumed in restaurants are generally higher in saturated fat, cholesterol and sugar. A transformation in the pattern of consumption has led to the creation of an obesogenic environment, and an increase in the prevalence of lifestyle diseases such as cardiovascular diseases and diabetes [10]. The share of daily calories consumed in restaurants and fast food establishments was reported to have increased from 6 to 20% between 1977 and 78 and 2005–08 [11].

Consumers’ knowledge of, and their ability to estimate, calorie count, and the fat, saturated fat, and sodium content of food was investigated by Burton et al., [10]. The actual fat and saturated fat levels were found to be higher than consumers’ estimates [12], and menus lack the information that gives consumers the capacity to choose more healthful foods.

The purpose of the present study is to identify the knowledge, attitudes, and practices of adults regarding the implementation of menu labeling in dine-in restaurants.

## Methods

The study was conducted in the cities of Sharjah and Dubai in the United Arab Emirates and approved by Ethical Committee at the University of Sharjah. Consent was obtained from participants before they were interviewed.

### Target population

A convenient sample of 2020 participants was collected from the two cities. The criterion for inclusion was being an adult male or female aged ≥ 18 years old. Data was collected using a questionnaire in different clusters in heavily populated areas. The questionnaires were distributed in public places such as parks, malls, educational institutes, neighborhoods, governmental buildings, and coffee shops in Sharjah and Dubai.

It is worth noting that UAE has a high entry rate record from secondary to higher education [13]. It was reported that about 95% of all girls and 80% of boys finished their secondary high school year to apply for admission to a higher education institution.

### Study design

The study was a quantitative, cross-sectional study that used a relevant validated questionnaire [4]. Some modifications to the questionnaire were made in order to identify the percentage of participants having nutritional awareness and who were interested in the implementation of calorie information on restaurant menus.

### Data collection

A pilot study using the questionnaire was conducted to assess if any changes were required. The main questionnaire was subsequently adjusted by adding a third option, “Neither”, to the question asking whether the participants favor or oppose the government mandating menu labeling

The questionnaire was tested for validity by a panel composed of four professionals in the Clinical Nutrition and Dietetics Department. The questionnaire was divided into four main sections: background information, knowledge, attitude and practices. The background information included age, gender, nationality, city of residence, and the frequency of dining outside the home. Participants’ knowledge was tested by asking about the estimated daily energy requirements for each gender and for their different physical activity levels (active and inactive). Attitude of the population sample measured the extent to which menu labeling was important to the participant, whether they supported menus being labeled, and if so, whether stamping or labeling menus with caloric values was preferred. Practices assumed that dine-in restaurant menus were labeled and participants were asked if they would choose labeled menu restaurants, select foods that are lower in calories, and whether their selection of foods was based on cost, appetite or caloric content.

The questionnaire was distributed along with the consent form and their contents were explained to the participants. The consent form, signed by the participants before completing the questionnaire, provided the option to accept or refuse participation in the study.

### Statistical analysis

Data was analyzed by using Statistical Package for the social sciences (SPSS) version 21.0(Statistical Package for Social Sciences, Version 21). Descriptive analysis, frequencies and percentages were calculated for demographic data. Chi-square test was used to study the correlation between two variables. Differences were considered significant at *p* < 0.05.

## Results

### Characteristics of the study sample

Approximately half of the respondents were females, under 24 years of age (60%), non-Emirati Arab (62%), living in Sharjah (52%), and had education more than a high school degree (76%). About one third (36%) of respondents reported visiting dine-in restaurants one to three times per month (Table 1).

**Table 1:** Frequency distribution of the general characteristics of the participants (*N* = 2020)

Variables	Percent	Number
Gender		
Male	43	870
Female	57	1146
Nationality		
Emirati	21	422
Non Emirati Arab	62	1237
Non Arab	17	333
Age		
18–24	60	1193
25–39	28	571
40+	12	241
Education Level		
High School or less	24	477
More than High school	76	1525
City of Residence		
Abu Dhabi	13	263
Dubai	24	485
Sharjah	52	1035
Other Cities	11	216
Restaurant Visit		
Never	3	68
1–3 times per month	36	715
1–2 times per week	35	700
3–4 times per week	16	329
Once Daily	10	195

### Knowledge of daily caloric requirements

Most of the respondents were able to identify the correct caloric intake for moderately active men (60%) and for moderately active women (59%) which, according to federal dietary guidelines, is between 1500 and less than 3000 kcal [14]. However, only 34% of participants were aware of the caloric requirements for inactive adults (Table 2).

**Table 2:** Percentage distribution of the participants who knew the caloric recommendations of the different levels of activities for the men and women (*N* = 2020)

Variables	Percent	Number
Moderately Active Men Caloric Recommendations		
Answered Correctly	60	1216
Answered Incorrectly	40	798
Moderately Active Women Caloric Recommendations		
Answered Correctly	59	1186
Answered Incorrectly	41	824
Inactive Adults Caloric Recommendations		
Answered Correctly	34	690
Answered Incorrectly	66	1319

The participants’ estimation of the correct daily energy requirements for moderately active men — between 1500-3000Kcal — differed significantly by gender, nationality, and education (*P < 0.05*). Similarly, estimations of the correct daily energy requirements for moderately active women (1500-3000Kcal) were significantly different by age (*P < 0.01*). Moreover, the correct estimation of energy requirements for inactive adults differed significantly by gender, nationality, and city of residence (*P <0.05*) (Table 3).

**Table 3:** Percentage of the participants who expressed knowledge of energy requirements of active and inactive men or women (*N* = 2020)

Knowledge of energy requirements												
Variable	Moderately active men				Moderately active women				Inactive adults			
	<1500	1500–3000	3000–4500	>4500	<1500	1500–3000	3000–4500	>4500	<1500	1500–3000	3000–4500	>4500
Age, Y												
18–24	(8.8%)	720 (60.4%)	343 (28.8%)	25 (2.1%)	340 (28.5%)	725 (60.8%)	113 (9.5%)	14 (1.2%)	638 (53.5%)	407 (34.1%)	101 (8.5%)	46 (3.9%)
25–39	62 (10.9%)	337 (59.1%)	156 (27.4%)	15 (2.6%)	191 (33.6%)	301 (52.9%)	66 (11.6%)	11 (1.9%)	310 (54.9%)	194 (34.3%)	43 (7.6%)	18 (3.2%)
40+	23 (9.7%)	153 (64.6%)	56 (23.6%)	5 (2.1%)	65 (27.7%)	151 (64.3%)	15 (6.4%)	4 (1.7%)	135 (56.7%)	85 (35.7%)	14 (5.9%)	4 (1.7%)
*P*-Value	0.542				0.016				0.516			
Gender												
Male	92 (10.6%)	550 (63.4%)	211 (24.3%)	15 (1.7%)	257 (29.8%)	497 (57.6%)	94 (10.9%)	15 (1.7%)	430 (49.8%)	319 (36.9%)	82 (9.5%)	33 (3.8%)
Female	101 (8.8%)	663 (58.1%)	349 (30.6%)	29 (2.9%)	342 (29.9%)	686 (60%)	101 (8.8%)	14 (1.2%)	656 (57.5%)	371 (32.5%)	79 (6.9%)	35 (3.1%)
*P*-Value	0.006				0.317				0.005			
Nationality												
Emirati	55 (13.1%)	249 (59.4%)	106 (25.3%)	9 (2.1%)	121 (28.9%)	242 (57.8%)	51 (12.2%)	5 (1.2%)	213 (50.7%)	152 (36.2%)	39 (9.3%)	16 (3.8%)
Non Emirati Arab	96 (7.8%)	746 (60.4%)	366 (29.6%)	28 (2.3%)	376 (30.5%)	623 (58.6%)	114 (9.2%)	21 (1.7%)	710 (57.6%)	388 (31.5%)	95 (7.7%)	40 (3.2%)
Non Arab	36 (10.9%)	206 (62.2%)	81 (24.5%)	8 (2.4%)	92 (28%)	205 (62.3%)	29 (8.8%)	3 (0.9%)	153 (46.5%)	142 (43.2%)	23 (7%)	11 (3.3%)
*P*-Value	0.025				0.431				0.003			
City of Residence												
Abu Dhabi	24 (9.1%)	160 (60.8%)	68 (25.9%)	11 (4.2%)	76 (28.9%)	148 (56.3%)	33 (12.5%)	6 (2.3%)	135 (51.3%)	89 (33.8%)	32 (12.2%)	7 (2.7%)
Dubai	38 (7.9%)	312 (64.5%)	125 (25.8%)	9 (1.9%)	135 (28%)	291 (60.4%)	53 (11%)	3 (0.6%)	249 (51.6%)	198 (41%)	24 (5%)	12 (2.5%)
Sharjah	100 (9.7%)	609 (59.1%)	302 (29.3%)	19 (1.8%)	326 (31.7%)	603 (58.6%)	86 (8.4%)	14 (1.4%)	575 (56%)	332 (32.3%)	80 (7.8%)	40 (3.9%)
Other Cities	25 (11.6%)	123 (56.9%)	62 (28.7%)	6 (2.8%)	58 (26.9%)	131 (60.6%)	21 (9.7%)	6 (2.8%)	124 (57.7%)	62 (28.8%)	21 (9.8%)	8 (3.7%)
*P*-Value	0.207				0.116				0.002			
Education												
High School or less	65 (13.7%)	275 (57.8%)	126 (26.5%)	10 (2.1%)	153 (32.2%)	264 (55.6%)	48 (10.1%)	10 (2.1%)	247 (52.2%)	159 (33.6%)	46 (9.7%)	21 (4.4%)
More than High school	124 (8.2%)	931 (61.2%)	431 (28.3%)	35 (2.3%)	440 (29%)	915 (60.3%)	144 (9.5%)	19 (1.3%)	837 (55.1%)	522 (34.4%)	113 (7.4%)	46 (3%)
*P*-Value	0.005				0.206				0.164			

### Attitudes towards menu labeling

The majority of the participants reported that menu labeling was very useful (47%) and somewhat useful (35%) (Table 4) and favored mandatory menu labeling (76%) (Table 5).

**Table 4:** Percentage distribution of the participants who expressed their views about the usefulness of menu labeling (*N* = 2020)

Variables	Percent	Number
Very Useful	47	941
Somewhat Useful	35	709
Not Very Useful	12	233
Not At All Useful	7	136

**Table 5:** Percentage distribution of the participants who expressed their views about the usefulness of menu labeling (*N* = 2020)

Variables	Percent	Number
Favor	76	1539
Oppose	7	131
Neither	17	343

Age, gender and nationality are shown to be significant variables associated with the participants’ attitudes towards menu labeling. In particular, young females of non-Emirati Arab nationality are seen to favor menu labeling in dine-in restaurants (*P* *<* *0.005*) (Table 6).

**Table 6:** Percentage distribution of the demographic variables of the participants as associated with attitude towards menu labeling (2020)

	Favor	Oppose	Neither
Variable	%	%	%
Age, Y			
18–24	72.2	7.5	20.3
25–39	81.9	5.6	12.5
40+	85	3.3	(11.7
*P*-Value	0.000		
Gender			
Male	73.1	7.3	19.7
Female	79	5.9	15
*P*-Value	0.007		
Nationality			
Emirati	76.8	8.8	14.5
Non Emirati Arab	76.9	5.8	17.3
Non Arab	74.3	5.4	20.2
*P*-Value	0.071		
City of Residence			
Abu Dhabi	79.8	5.7	14.4
Dubai	74.8	5.8	19.3
Sharjah	75.7	7.1	17.2
Other Cities	80.9	5.1	14
*P*-Value	0.353		
Education			
High school or less	74.7	6.8	18.6
More than High school	77.2	6.3	16.5
*P*-Value	0.520		

### Future practices towards menu labeling

When participants’ were asked about the likelihood of dining at a menu-labeled restaurant, 48% replied that they will more likely, to choose to dine in a restaurant that has menu labeled (Table 7).

**Table 7:** Percentage distribution of the participants who are likely to eat at a labeled restaurant (*N* = 2020)

	Percent	Number
Variable		
More Likely	48	961
Less Likely	24	486
Neither	28	570

The association between demographic variables of the participants and their likeliness to eat at a menu-labeled dine-in restaurant is shown. Significant differences by age, city of residence and education (*P < 0.005*) were reported for these practices (Table 8).

**Table 8:** Percentage distribution of the demographic variables of the participants as associated with their likeliness towards dining in a menu labeled restaurant (2020)

	More Likely	Less Likely	Neither
Age, Y			
18–24	507 (42.5%)	315 (26.4%)	370 (31.0%)
25–39	308 (54.0%)	120 (21.1%)	142 (24.9%)
40+	137 (57.1%)	47 (19.6%)	56 (23.3%)
*P*-Value	0.000		
Gender			
Male	395 (45.5%)	213 (24.5%)	260 (30.0%)
Female	564 (49.3%)	271 (23.7%)	310 (27.1%)
*P*-Value	0.217		
Nationality			
Emirati	205 (48.6%)	100 (23.7%)	117 (27.7%)
Non Emirati Arab	596 (48.2%)	304 (24.6%)	336 (27.2%)
Non Arab	145 (43.8%)	72 (21.8%)	114 (34.4%)
*P*-Value	0.135		
City of Residence			
Abu Dhabi	112 (42.7%)	84 (32.1%)	66 (25.2%)
Dubai	238 (49.1%)	95 (19.6%)	152 (31.3%)
Sharjah	496 (48.0%)	241 (23.3%)	297 (28.7%)
Other Cities	107 (49.8%)	52 (26.5%)	51 (23.7%)
*P*-Value	0.007		
Education			
High school or less	205 (43.0%)	116 (24.3%)	156 (26.7%)
More than High school	750 (49.3%)	365 (24.0%)	407 (28.2%)
*P*-Value	0.022		

## Discussion

This study has found that most participants estimated correctly the energy requirements for moderately active men and women, but tended to underestimate energy requirements for inactive adults. They expressed a positive attitude towards menu labeling in dine in restaurants; a majority viewed it as very or somewhat useful, reported being more likely to eat in a restaurant with menu labeled restaurant.

The findings of this study were consistent with those of Bleich & Pollack [4], who found that the American adult population was well informed about energy requirements for moderately active men and women, but tended to underestimate energy requirements for inactive adults. That study also reported that Americans expressed a positive attitude towards calorie posting in chain restaurants; a majority viewed it as very useful or somewhat useful. In the current study, the majority of the participants favored menu labeling, stating that having menus provide caloric labeling in dine–in restaurants is very useful.

The results of this study also showed a positive correlation between age and likeliness to eat at a menu-labeled restaurant, with the majority of the young participants responded positively to menu-labeling. Moreover, it was found that participants who had more than high school education answered the daily caloric requirement questions correctly. Krukowski et al. [15] has reported that college students want nutrition labels and would use them to inform their food purchasing decisions.

In our study, the majority of the participants, and women in particular, had a favorable response to menu labeling and correctly estimated the caloric requirements for active women and men. The majority of the respondents in this study expressed their intention to choose to eat at a menu-labeled restaurant. Similarly, Din [16] showed that that women were more interested in menu labeling, and believed that it aid in regulating their intake than men.

It is worth noting that the majority of the respondents in this study eat more frequently at restaurants. Mussaiger [17] had reported that the proportion of obesity increased to reach 52.7% among those who eat outside the home for more than 5 times per week since the foods are eaten outside the home is more likely to be high in total energy, total fat, saturated fat.

Several prospective studies have reported that eating more frequently away from home in restaurants, is associated with weight gain over time compared to infrequent restaurant eating [18, 19]. Therefore, a better understanding of public perceptions about calorie posting may encourage policy makers to adopt this low-cost policy tool in order to educate consumers to make healthier food choices.

It has been reported that people who used menu labeling to determine calorie content consumed significantly fewer calories during a meal compared with people who did not use menu labeling [20]. Roberto et al. [7] studied the impact of menu labeling on food choices and intake and found that research participants who received caloric-labeled menus consumed 14% fewer calories than those who received menus without calorie labeling. An average reduction of 100 cal per meal resulted among restaurant patrons who ordered reduced-calorie meals in response to calorie postings on the menus [21].

To our knowledge, the current study is the first to assess consumers’ understanding of overall daily energy requirements and the perceived effectiveness of calorie posting in dine–in restaurants in the UAE. The results from this study may form the basis of future strategies in mandating calorie posting in dine-in restaurants as well as fast food chains in the United Arab Emirates.

Furthermore, menu-labeling may serve to encourage restaurants to highlight lower calorie options and/or introduce healthier options. Given the positive findings of intention to choose restaurants with labeled menus, some restaurants may also begin providing caloric information voluntarily.

This study has some limitations including, firstly, that as a cross-sectional study, it only allows associations to be addressed. Second, as Bleich & Pollack [4] stated, the range of calories in the correct response category for the caloric knowledge question was broad; thus, the finding of relatively high caloric knowledge may be biased upwards. Third, given that the correct answer to the caloric knowledge questions was the same for all groups (e.g. moderately active men, moderately active women, and inactive adults), some participants may have assumed that the answer must change across groups. This would bias our results downwards and may partially explain our finding of low caloric literacy about inactive adults.

## Conclusion

The results of this study encourage further research concerning the implementation of menu labeling in restaurants. This is especially significant given that this is a new concept in the UAE. Additionally, research studies of nutrient and calorie awareness are needed to ensure that UAE residents understand calorie requirements and how to read and apply nutrition facts on product labels to their consumption practices. It would also be of interest to conduct an experimental study comparing two restaurants − one with menu labeling and one without − by measuring the amount of calories purchased by individuals. Studies should identify how and where menu labeling can be best presented to most effectively help consumers make healthier choices and lower their caloric intake.

Given that there is an increasing frequency of restaurant visitors in UAE, mandating calorie posting in dine-in, as well as fast food restaurants may be a useful policy tool for promoting appropriate energy intake, to help consumers make food choices in restaurants, contributing to lower rates of obesity.
